# Author Self-Citation in the General Medicine Literature

**DOI:** 10.1371/journal.pone.0020885

**Published:** 2011-06-16

**Authors:** Abhaya V. Kulkarni, Brittany Aziz, Iffat Shams, Jason W. Busse

**Affiliations:** 1 Child Health and Evaluative Sciences, The Hospital for Sick Children, Toronto, Ontario, Canada; 2 Institute for Work and Health, Toronto, Ontario, Canada; 3 Department of Clinical Epidemiology and Biostatistics, McMaster University, Hamilton, Ontario, Canada; Children's Hospital of Eastern Ontario, Canada

## Abstract

**Background:**

Author self-citation contributes to the overall citation count of an article and the impact factor of the journal in which it appears. Little is known, however, about the extent of self-citation in the general clinical medicine literature. The objective of this study was to determine the extent and temporal pattern of author self-citation and the article characteristics associated with author self-citation.

**Methodology/Principal Findings:**

We performed a retrospective cohort study of articles published in three high impact general medical journals (*JAMA*, *Lancet*, and *New England Journal of Medicine*) between October 1, 1999 and March 31, 2000. We retrieved the number and percentage of author self-citations received by the article since publication, as of June 2008, from the Scopus citation database. Several article characteristics were extracted by two blinded, independent reviewers for each article in the cohort and analyzed in multivariable linear regression analyses. Since publication, author self-citations accounted for 6.5% (95% confidence interval 6.3–6.7%) of all citations received by the 328 articles in our sample. Self-citation peaked in 2002, declining annually thereafter. Studies with more authors, in cardiovascular medicine or infectious disease, and with smaller sample size were associated with more author self-citations and higher percentage of author self-citation (all p≤0.01).

**Conclusions/Significance:**

Approximately 1 in 15 citations of articles in high-profile general medicine journals are author self-citations. Self-citation peaks within about 2 years of publication and disproportionately affects impact factor. Studies most vulnerable to this effect are those with more authors, small sample size, and in cardiovascular medicine or infectious disease.

## Introduction

Citation counts received by journal articles are used to inform decisions of academic promotion and in the assessment of research and journal impact. Author self-citation, as opposed to journal self-citation, occurs when authors reference their own publications and this practice can be regarded positively (e.g., guiding readers to important relevant research) or negatively (e.g., intentionally inflating the impact of one's own work). Regardless of the motivation that underlies self-citation [1], there is only a limited body of literature that has addressed its quantitative impact in the general clinical medicine literature. Some studies [2], [3] have examined *synchronous* self-citation [4], in which the references of an index article are reviewed for previous works by the same author(s). By examining only the bibliographies of index articles, however, synchronous self-citation does not tell us about the importance of self-citation on the citation counts of the index articles, which is how the impact of articles and journals is commonly assessed. Therefore, to understand the impact of self-citation on citation counts, information is needed about *diachronous* self-citation [4], in which a citation database is used to establish when an index article is cited by future publications from the same author(s). Diachronous self-citation contributes directly to the overall citation count and could alter perceptions about the impact of an article or journal in which it appears. Glanzel et al., in a “macro level” analysis [5], showed that diachronous self-citations occur earlier after publication than non-self citations, but they did not explore associations between individual article characteristics and self-citation. Other studies of diachronous self-citation are limited by narrow clinical focus [6], narrow geographical focus [7], or limited post-publication windows [6], [8]. We used Scopus (Elsevier), a relatively new citation database that includes a feature to isolate author self-citations from total citation counts, to examine diachronous self-citation in a large cohort of articles published in high-profile general medicine journals over 8 years ago. We were specifically interested in identifying the relative contribution of author self-citation to the overall citation count of articles and to determine if specific article characteristics were associated with author self-citation.

## Methods

Through hand-searching, we acquired a sample of original research papers published in *JAMA*, *Lancet*, and *New England Journal of Medicine (NEJM)* between October 1, 1999 and March 30, 2000 [9], [10]. This period of publication is well within the coverage range of Scopus [11]. We included all articles published under the following table of content headings: “Original Contributions” in *JAMA*, “Original Research–Articles” in *Lancet*, and “Original Articles” in *NEJM*.

For each article two reviewers (AVK and JWB) trained in health research methodology extracted independently and in duplicate, the following characteristics, as previously described, [9], [10]: 1) the journal in which the article appeared (*JAMA*, *Lancet*, or *NEJM*); 2) study design (randomized trial, prospective observational study, retrospective study, meta-analysis, or survey study); 3) clinical category of the article (cardiovascular, general medicine, infectious disease, obstetrics/gynaecology, oncology, or other); 4) whether the author by-line for the article included group authorship; 5) the number of individual authors named in the top author by-line excluding group names; if the group name was the only name listed, then this was counted as 1 author only; 6) whether the research was performed partly or fully in the United States (meaning that research participants were recruited within the United States or, for research that did not use research participants, e.g., meta-analyses, the address of the corresponding author was within the United States); 7) sample size of the study (for meta-analyses, the sample size was taken as the total number of patients in all analyzed studies); 8) declared for-profit industry funding; 9) if the article studied a drug or medical device; and 10) if the study had been reported by the Associated Press in the news media, based on a contemporaneous daily search of the Associated Press news wire during the 6 month publication period of our sample. Reviewers resolved discrepancies by discussion.

In June 2008, after a minimum of 2 hours training in the use of Scopus (Elsevier), two of us (BA, IS) determined citation counts for each article according to this citation database. Currently, there are at least three online databases available to track citation counts of articles: Web of Science, Scopus, and Google Scholar. We have previously shown in this cohort of articles that Scopus retrieved more citations, on average, than Web of Science (Thomson Reuters), and both had greater total citation accuracy than Google Scholar [9]. We determined for each full year since publication (i.e., 2001 to 2007, inclusive) and in total since publication: total overall citations received, total author self-citations received (which Scopus determines using their Author Identifier algorithm to identify and track unique authors), total non-self-citations, and the percentage of total citations that were author self-citations. We also recorded the total number of journal self-citations, defined as a citation received from an article published in the same journal as the index article.

Two of us (AVK, JWB) performed repeat, independent citation searches for the first 30 articles in our cohort (based on their chronological order of publication) and on another 50 articles, randomly selected, to confirm accuracy of data collection.

We assessed the accuracy of Scopus' determination of author self-citation. For a randomly selected sample of 20 articles we identified all citations and author self-citations identified by Scopus and reviewed the author by-line for each citing article to determine the number of false negative self-citations (true author self-citations that were not identified as self-citations by Scopus) and false positive self-citations (non-self-citations that were identified as self-citations by Scopus). From this we calculated the sensitivity and specificity of Scopus in identifying self-citations.

### Statistical Analysis

To explore the association between article characteristics and author self-citation we performed a linear regression analysis with total author self-citation count per article since publication as the dependent variable. The following independent variables (as described above) were entered into a single multivariable model: 1) number of authors appearing in the author byline (excluding group names); 2) non-self-citation count (divided into quintiles); 3) journal of publication; 4) whether the study was randomized; 5) clinical category of the article; 6) group authorship; 7) whether the research was performed partly or fully in the United States; 8) sample size of the study (log10-transformed); 9) declared for-profit industry funding; 10) whether the article studied a drug or medical device; 11) contemporaneous reporting by the Associated Press. Variance inflation factors for all variables were less than 5 indicating no worrisome multicollinearity [12]. To explore the relative contribution of self-citation to the total citation count, we repeated this analysis using percent author self-citation per article as the dependent variable. Because of the skewed non-normal distribution of self-citation count per article and percent self-citation per article, we log10-transformed these data for regression analyses. The approximation to the normal distribution was confirmed with a Kolmogorov-Smirnov test (p>0.08) and examination of probability-probability plots. Because some articles had zero self-citations we added a value of “1” to self-citation counts and 1% to the percent self-citations of all articles prior to log10-transformation. All p-values of <0.05 were considered statistically significant. All analyses were performed with SPSS Advanced Statistics 17.0 (SPSS Inc., Chicago, IL).

## Results

Characteristics of the 328 index articles are shown in Table 1. The median sample size for these articles was 642 (interquartile range = 147 to 6363) and the median number of authors was 5 (interquartile range = 4 to 9). In a random sample of 20 of the 328 articles, the sensitivity of Scopus in accurately identifying author self-citations was 95.5% and the specificity was 100% (Scopus identified 298 self-citations, of which none were false-positive, and 3601 non-self-citations, of which 14 were false-negative).

**Table 1:** Characteristics of the index article cohort.

*Variable*	*Number of articles*
**Journal of publication**	
- JAMA	100
- Lancet	126
- NEJM	102
**Declared industry funding**	
- yes	82
- no	246
**Study of a drug or medical device**	
- yes	102
- no	226
**Clinical category**	
- cardiovascular	57
- general medicine	29
- oncology	30
- infectious disease	62
- obstetrics & gynaecology	25
- other	125
**Group authorship**	
- yes	68
- no	260
**News media coverage of article**	
- yes	97
- no	231
**Location of study**	
- partly/exclusive in United States	177
- not in United States	151
**Study design**	
- randomized	92
- prospective	108
- retrospective	92
- meta-analysis	15
- survey	19

**Figure 1:**
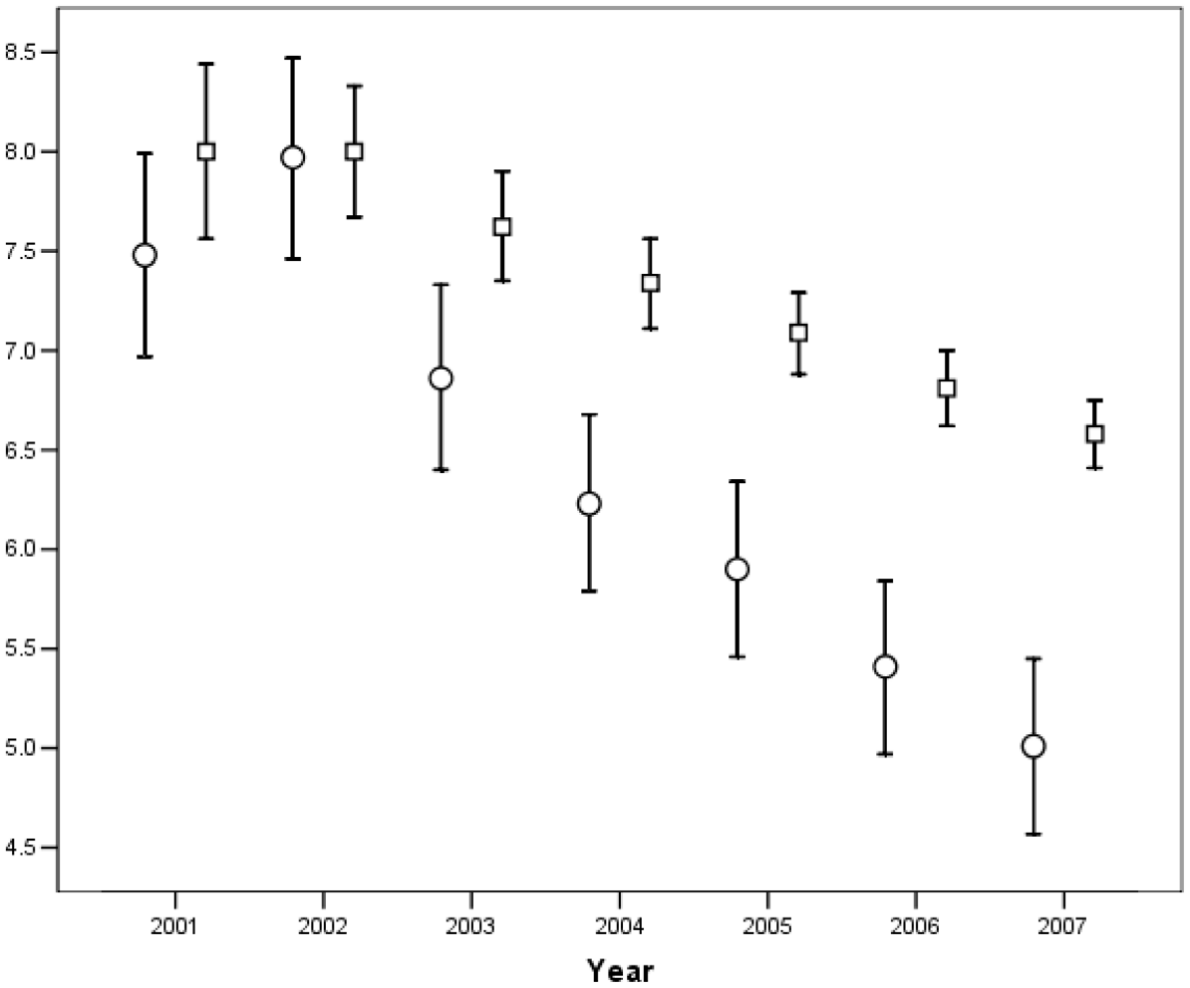
Graph showing the annual percent author self-citation (circles) and the cumulative percent author self-citation since publication (squares) for each full calendar year since publication. The bars represent the associated 95% confidence intervals.

Since publication, the 328 articles in our cohort received 82183 citations of which 5355 were author self-citations (6.5%, 95% confidence interval 6.3–6.7%) and 879 were journal self-citations (1.1%, 95% CI 1.0–1.1%). The year-by-year and cumulative percent author self-citation, beginning in 2001 (the first full year since publication of all articles) is shown in Figure 1.

Since publication, the median number of self-citations received was 11 per article (interquartile range 4–23, mean 16.0) and this accounted for a median of 6.4% of all citations per article (interquartile range 2.8–11.3, mean 8.4). In our regression analysis (Table 2), the following characteristics were associated with more *total author self-citations per article*: more authors, more non-self-citations, studies in cardiovascular medicine or infectious disease, publication in *JAMA*, and smaller sample size. The adjusted R-squared for the model was 0.45. In a separate regression model (Table 2), the following characteristics were associated with a *higher percentage of author self-citation per article*: more authors, studies in cardiovascular medicine or infectious disease, and smaller study sample size. The adjusted R-squared for this model was 0.16.

**Table 2:** Results of regression analyses.

Characteristic of Interest	Total self-citation count* (unstandardized regression coefficients (95% CI))	p-value	Percentage of self-citation* (unstandardized regression coefficients (95% CI))	p-value
Number of authors	0.02 (0.01 to 0.03)	<0.001	0.02 (0.01 to 0.03)	<0.001
Non-self-citations		<0.001		NS
- quintile 1	reference			
- quintile 2	0.26 (0.14 to 0.39)			
- quintile 3	0.39 (0.26 to 0.52)			
- quintile 4	0.55 (0.42 to 0.68)			
- quintile 5	0.81 (0.67 to 0.96)			
Subject category		0.005		0.01
- infectious disease	0.14 (0.03 to 0.25)		0.14 (0.03 to 0.26)	
- cardiovascular	0.22 (0.11 to 0.34)		0.20 (0.08 to 0.32)	
- oncology	0.13 (−0.02 to 0.28)		0.09 (−0.06 to 0.23)	
- general medicine	0.16 (0.00 to 0.32)		0.13 (−0.03 to 0.28)	
- obstetrics/gynaecology	0.09 (−0.07 to 0.25)		0.12 (−0.04 to 0.27)	
- other	reference		reference	
Journal		0.03		NS
- NEJM	0.11 (−0.01 to 0.23)			
- JAMA	0.18 (0.05 to 0.31)			
- Lancet	reference			
Sample size, log-transformed	−0.08 (−0.12 to −0.04)	<0.001	−0.08 (−0.12 to −0.04)	<0.001

95% CI = 95% confidence interval; NS = not significant (data not shown).

*Each model also included several non-significant variables, as described in the text (not shown in the table). All unstandardized regression coefficients represent the expected change in the dependent variable (on the log10 scale) associated with the independent variable of interest. Therefore, a coefficient value of 0.1 represents 25% increase, 0.2 represents 58% increase, 0.3 represents 100% increase, and 0.4 represents 150% increase.

## Discussion

In a cohort of articles published in high-profile general medicine journals, we found that, over an 8 year follow-up period, approximately 6.5% (95% CI 6.3–6.7) of citations received were author self-citations and 1.1% (95% CI 1.0–1.1) were journal self-citations. Author self-citations peaked about two years after publication and then declined progressively thereafter (Figure 1). Studies with more authors, those in cardiovascular medicine or infectious disease, and those with a smaller sample size were associated with more author self-citations and higher percent of author self-citation per article. Publication in *JAMA* and more non-self-citations were also associated with more total author self-citations per article.

Strengths of our study include the broad range of articles included in our cohort and the long post-publication window, which allowed for a meaningful assessment of the temporal pattern of citations. Our multivariable analysis was adjusted for several potential confounders and explained a large portion of the variance in self-citation count (adjusted R-squared 0.45). We used Scopus' Author Identifier to determine self-citations, which uses a complex algorithm to distinguish and identify unique authors. Elsevier claims that this algorithm has an accuracy of 99% (http://www.info.sciverse.com/documents/files/scopus-training/resourcelibrary/pdf/br_author_identifier.pdf, last accessed April 22, 2011), and we found greater than 95% sensitivity and perfect specificity in our validation exercise. We do, however, recognize limitations in our work. Our study only quantified self-citations, without assessing context. There are numerous possible reasons for self-citation that include referencing previous relevant work (as we have done in this paper, for example), increasing a sense of the author's mastery of the subject, raising the profile of earlier work, correcting earlier work, and inflating the citation profile of earlier work [1]. Our study was limited to high-profile general medical journals and our results may have limited generalizability to other literature. For example, lower profile journals in focused subspecialties tend to have higher percentage of journal self-citation [13]. As well, the regression models we developed have not been externally validated in an independent sample of index articles.

The existing literature on diachronous author self-citation is sparse. In a study of 289 diabetes articles with a post-publication follow-up of approximately 2 years, Gami et al. found that the mean percentage of author self-citations was 18% [6], much higher than in our study (8.4%). Their median self-citation percentage (7%), however, was similar to ours (6.4%), suggesting that their mean value was skewed by outliers. In a study of scientific papers originating only from Norway over a 15-year period, Aksnes et al. found that the percentage of self-citation was higher in multi-authored papers and in otherwise poorly cited papers [7]. Glanzel et al. reported on a 9 year aggregate analysis of articles listed in the citation database Web of Science and found that the peak in self-citations occurred earlier than non-self-citations [5]. Davarpanah and Amel confirmed these findings in a similar study using a 3 year post-publication window [8]. They also found that in general and internal medicine, self-citation accounted for about 16% of all citations, lower than organic chemistry, plant sciences, and electronic engineering. Neither of these studies assessed the influence of individual article characteristics on self-citation. To our knowledge, ours is the first study to provide a detailed report of author self-citation rates for general medicine articles covering a wide range of subspecialties and countries, with a long post-publication follow-up, and including details about individual article characteristics.

Our findings have important implications for the use and interpretation of citation counts. Self-citations, which peaked within 2 years of publication, disproportionately affect journal impact factor, which is based on a post-publication window of no more than 2 years [14]. The early concentration of self-citations might, at least partially, be the results of authors having advanced knowledge of their own works before they are actually published and publicly available. In our analysis, smaller studies, with greater numbers of authors, in cardiovascular medicine or infectious disease were associated with greater subsequent percentage of author self-citations and more total author self-citations. While it seems intuitive that more authors would result in more self-citations, the explanation for the effect of subspecialty and study sample size are not clear. The differences in self-citations among medical subspecialties might be explained by different overall citation patterns and speed of citation in those specialties.

Because impact factor includes self-citations, it measures not only the degree to which articles in a journal are cited by others, but also the degree to which authors of the index article publish more, similar works that cite the index article. One could argue that self-citations should legitimately be included in impact factor because a self-citation contributes just as much to increasing the exposure of an index article (and journal) as a non-self-citation. Removing self-citations from the impact factor, however, would more accurately reflect how other researchers perceive the index article. If self-citations were removed from impact factor calculations, we would expect that the high-profile journals used in our sample would see a decrease in their impact factor of approximately 8% - the approximate percent of self-citations in the first 2 years after publication (slightly more so for *JAMA* and slightly less so for *Lancet*). Lower-profile journals, which probably have higher percentages of self-citation, would likely see an even larger drop in their impact factor. This could potentially alter the rankings of journals within specialities (these rankings are advertised on some journal websites). In our sample, for example, the 2002 impact factor for *NEJM* was 31.736, for *JAMA* it was 16.586, and for *Lancet* it was 15.397. Removing self-citations might have switched the rankings of *JAMA* and *Lancet*, since *JAMA* articles had more self-citations than *Lancet*. Alternatively, calculating impact factors over a larger time window, beyond 2 years, would also attenuate the influence of self-citations.

In summary, our analysis found that author self-citations account for approximately 1 in 15 citations received by articles published in high-profile general medical journals over an 8 year post-publication period. Self-citations account for a greater percentage of citations early after publication, progressively decreasing after 2 years. Certain article characteristics are associated with increased self-citation, and should be taken into account when assessing citation counts for individual articles or journal impact factors. Future research should explore the context of self-citations in order to assess the validity of this common practice.
